# Systematic Evaluation of Antioxidant Efficiency and Antibacterial Mechanism of Bitter Gourd Extract Stabilized Silver Nanoparticles

**DOI:** 10.3390/nano11092278

**Published:** 2021-09-02

**Authors:** Kavya Moorthy, Kai-Chih Chang, Wen-Jui Wu, Jun-Yi Hsu, Po-Jen Yu, Cheng-Kang Chiang

**Affiliations:** 1 Department of Chemistry, National Dong Hwa University, Hualien 974301, Taiwan; kavyamoorthykali@gmail.com (K.M.); 410612017@gms.ndhu.edu.tw (J.-Y.H.); 2 Department of Laboratory Medicine and Biotechnology, Tzu Chi University, Hualien 97004, Taiwan; kaichih@gms.tcu.edu.tw (K.-C.C.); w200811@gms.tcu.edu.tw (W.-J.W.); 107323110@gms.tcu.edu.tw (P.-J.Y.); 3 Department of Laboratory Medicine, Buddhist Tzu Chi General Hospital, Hualien 97004, Taiwan

**Keywords:** bitter gourd extract, green synthesis, silver nanoparticles, antibacterial, antioxidant, drug-resistant bacteria

## Abstract

In this study, we accentuate the facile and green synthesis of ecologically viable silver nanoparticles (AgNPs) using aqueous (A-BGE) and ethanolic (E-BGE) dried bitter gourd (Momordica charantia) fruit extract as reducing and capping agents. Although AgNPs synthesized using BGEs have been reported earlier in fundamental antimicrobial studies, the possible antioxidant activity, antibacterial efficacy against superbugs, and a potential antimicrobial mechanism are still lacking. The characterization of as-prepared AgNPs was studied through UV-vis, TEM, Zeta-potential, FT-IR, XRD, and XPS analysis. The antioxidant ability of BG-AgNPs was extensively evaluated through DPPH and FRAP assays, which showed that A-BG-AgNPs possessed higher scavenging ability and superior reducing power due to the high phenolic content present in the BG extract. Furthermore, A-BG-AgNPs were highly stable in various physiological media and displayed excellent antibacterial activity against drug-resistant bacterial strains (i.e., MIC value of 4 µg/mL). The generation of reactive oxygen species evidenced that the possible antimicrobial mechanism was induced by BG-AgNPs, resulting in bacterial cell damage. Within the minimal hemolysis, the BG-mediated AgNPs possessed synergistic antioxidant and antibacterial agents and open another avenue for the inhibition of the growth of pathogens.

## 1. Introduction

Nanotechnology is an innovative field offering an opportunity to pioneer a method of treating microbial infection through nanoparticles [1]. These nanomaterials, being ultrafine particles with specified physiochemical features rather than bulk materials that act as drug vectors fighting against drug-resistant pathogens, can exert multiple antibacterial mechanisms [2]. The nanoparticles can act as antimicrobial agents, themselves, and as carriers for antibiotics with sustained, controlled bio-distribution with fewer side effects combating globally problematic multi-drug resistant (MDR) bacteria; thus, they are typically termed as nanoantibiotics. They may also be referred to as nanobactericides or nanocarriers depending on their action in terms of antibacterial activity, such as CuO, CeO_2_, MgO, AgNPs, AuNPs, NiNPs, TiO_2_, and ZnO, as per the literature. Silver nanoparticles have gained boundless interest due to the fact of their varying size, stability, and release of silver ions (Ag^+^), thereby interacting with the bacterial cell wall, generation of reactive oxygen species (ROS), and interaction with nucleic materials, leading to cell death compared to other metallic nanoparticles [3,4,5]. AgNPs are also widely used in wastewater treatment, the textile industry, and in biomedical [6,7] and biosensor applications [8]. Due to the emergence of the COVID-19 pandemic [9], recent studies have focused on the antiviral ability of AgNPs. However, bare AgNPs are prone to agglomeration and inferior storage stability, which makes them limited for use in practical applications, as they decrease in activity over time. Several nanocomposites have been prepared in this concern by attaching AgNPs to inorganic or organic materials. Mesoporous silica is notable and most efficient due to the fact of its high surface area, biocompatibility, and easy surface modification [10,11]. These nanocomposites can be prepared through chemical, physical, and biological [6] methods. However, the conventional techniques involved in the processing of nanomaterials are eco-unfriendly and utilize toxic chemicals.

With the furtherance of science, plant-derived extracts, including those from leaves, bark, seeds, peels, pods, stems, callus, petals, rhizomes, gums, roots, and fruit, have been used as sources for the reduction in metallic precursor to nanoparticles [12]. This is mainly due to biomolecules, such as phenolics, alkaloids, flavonoids, steroids, and terpenoids, present in plants that can act as stabilizing and capping agents [13]. Several previously reported plant-derived mediated AgNPs, including *Terminalia arjuna*, *Pimpinella anisum*, *Nyctanthes arborists*, *Potentilla fulgens*, *Rosa canina*, *Styrax benzoin*, *Prosopis juliflora* and *Fagus sylvatica*, have exhibited antibacterial activities [12,14]. Being more biocompatible, environmentally sustainable, and cost effective, these biosynthesized nanoparticles reveal a wide range of applications in catalysis, bio-sensing, cancer therapy, antimicrobials, and drug delivery [15,16,17].

*Momordica charantia*, known as bitter melon, balsam pear, or bitter gourd (BG), is a widely cultivated indigenous medicinal and edible plant in tropical and subtropical regions worldwide [18]. It has been used in numerous traditional and folk medicines due to the fact of its pharmaceutical and nutraceutical phytochemicals attributed to every part of the plant, especially the fruits. The intensely bitter-tasting fruit with high antioxidant properties is considered a blood purifier for diabetes mellitus with fewer side effects [19]. Apart from this, BG also possesses other biological properties such as anti-cancer [20], anti-viral, antimicrobial [21], anti-inflammatory [22], hypotensive, and resisting obesity [23]. The major phytoconstituents responsible for the pharmaceutical properties are phenolic compounds (phenolic acids, flavonoids, and phenylpropanoids), cucurbitane-type triterpenoids, fatty acids, phytosterols, carotenoids, alkaloids, essential oil, saponins, proteins, polypeptides, lipids, carbohydrates, glycosides, and amino acids [24,25,26]. To our knowledge, extracts from bitter gourd leaves, stems, fruits, and peels were used to synthesize metallic nanoparticles such as gold (AuNPs) [27], silver (AgNPs) [28,29,30,31,32], zinc oxide (ZnONPs) [33], cerium oxide (CeO_2_NPs) [34] and copper (CuNPs) [35] nanomaterials. It is also notable that most of the abovementioned BG fruit extracts employed were prepared using fresh fruits via the decoction method [28,29,30,31], whereas Shanker et al. used dried ones through the Soxhlet extraction strategy [34]. Although several antibacterial studies have been conducted on bitter gourd extracts synthesized nanomaterials, the potential bactericidal mechanism and the antibacterial effectiveness against drug-resistant bacteria still remain unclear.

Herein, we introduce aqueous and ethanolic extracts from dried bitter gourd fruit (A- and E-BGE) for the green synthesis of AgNPs. We investigated the antioxidant potential and antibacterial activity of BG-medicated AgNPs (A- and E-BG-AgNPs) against several pathogenic drug-resistant and wild-type bacterial strains. The antioxidant ability of as-prepared AgNPs was also evaluated using the DPPH and FRAP assays. To further understand the antibacterial efficiency and possible mechanism of BG-mediated AgNPs, minimum inhibition concentration (MIC) test, instant antibacterial activity assay, ROS measurement, and electron microscopy experiments were conducted in this study.

## 2. Materials and Methods

### 2.1. Chemicals and Reagents

All chemicals and reagents were used in the experiment were of analytical grade and used without further purification. Silver nitrate (AgNO_3_, 99.85%), aluminum chloride (AlCl_3_, 99%), quercetin hydrate 95.0%, and potassium bromide (KBr, 99%) were obtained from Acros Organics (Geel, Belgium). Diphenyl-1-picrylhydrazyl (95%) and sodium carbonate were obtained from Alfa Aesar (Lancashire, UK). Gallic acid, sodium borohydride (NaBH_4_, 98%), and Folin-Ciocalteu phenol reagent were purchased from Sigma (St. Louis, MO, USA). The apparatuses and glassware were cleaned with aqua-regia and rinsed several times with distilled water followed by deionized (DI) water.

Bacterial strains, such as *E. coli* (ATCC 25922), *P. aeruginosa* (ATCC 27853), *A. baumannii* (ATCC 17978), and *S. aureus* strain (ATCC 25923) were obtained from the American Type Culture Collection (ATCC) and cultured according to the guidelines of the Clinical and Laboratory Standards Institute, respectively. Drug-resistant Gram-negative bacterial strains, including colistin- (CR32X 17978) and imipenem-resistant *A. baumannii* (IMP32X 17978), were induced manually until the concentration of antibiotic reached 32 µM, at Tzu chi university, respectively. This study was conducted according to the guidelines of Clinical and Laboratory Standards Institute, and the experiments associated with human blood collection and applications were approved by Tzu Chi Hospital (No. IRB105–146A).

### 2.2. Preparation of Bitter Gourd Extracts

Bitter gourd fruit (BG) in the mature stage was collected from the local market in Shoufeng county, Hualien, and powdered using a commercially available electric blender (250 W). Aqueous and ethanolic extracts of BG (A-BGE and E-BGE) were prepared by a slight modification of previously reported methods [36,37]. In brief, 2 g of dried BG powder in 100 mL of DI and 80% ethanol decocted at 70 °C for 30 min under magnetic stirring. After cooling to room temperature, the extracts were filtered through Whatman No. 1 filter paper prior to storage at 4 °C for further use.

### 2.3. Biosynthesis of Silver Nanoparticles Using Extracts

Typically, 10 mL of respective BG extract, including A-BGE and E-BGE, was added to 90 mL of aqueous silver nitrate solution (5 mM) in the dark under vigorous stirring and refluxed at 100 °C for 2 h. After the color of the reaction solution changed, the obtained solution was purified by centrifugation at 15,000 *g* for 30 min several times using DI water. The purified AgNPs were named as A-BG-AgNPs for aqueous extract, whereas E-BG-AgNPs were for ethanolic sections. These purified BG-AgNPs were dried using miVac Duo concentrator (Genevac Ltd., Ipswich, UK) at room temperature. To understand the influence of temperature for the synthesis of BG-AgNPs, AgNO_3_ solution was mixed with respective BG extract and refluxed at 60 or 200 °C for 6 h or 15 min, respectively.

### 2.4. Characterization of BG Mediated AgNPs

SpectraMax iD3 microplate reader (Molecular Devices, San Jose, CA, USA) was used to measure the UV-Vis absorbance spectra of BG-AgNPs. By using Spectrum one FT–IR Spectrometer (Perkin Elmer Instruments, Waltham, MA, USA), the FT-IR measurements for the respective BG extracts and BG-AgNPs were recorded between 4000 and 400 cm^−1^ using KBr as a reference. D2 phaser diffractometer (Bruker, Germany) with Cu K_α_ radiation with λ = 1.54 Å was utilized to analyze the powder X-ray diffraction pattern (P-XRD) of as-prepared AgNPs. K-Alpha X-ray photoelectron spectrometer (Thermo Scientific, Waltham, MA, USA) using a high-resolution monochromatic Al Kα line as excitation source was used in this study. Zeta Sizer Nano ZS (Malvern Instruments Ltd., Worcestershire, UK) was used for measuring the Zeta potential for the obtained BG-AgNPs (*n* = 3).

### 2.5. Phytochemical Analysis of BG Extracts

Two types of as-prepared BG extracts for about 5 mL were dried using miVac Duo concentrator. In total, 2 mg of the obtained dried BG extract was suspended in 1 mL of methanol and sonicated for 45 min. After centrifugation at 1000× *g* for 10 min, the supernatant was collected for the following assays.

#### 2.5.1. Total Phenolic Assay and Total Flavonoid Assay of BGs

Folin-Ciocalteu assay was undertaken to quantify the total phenolic content (TPC) in the bitter gourd extracts. A portion of about 0.08 mL of respective extracts and gallic acid for standard solution (5–500 μg/mL), DI water (0.24 mL), and (1:1) Folin-Ciocalteu’s phenol reagent (0.08 mL) was mixed at first, followed by saturated sodium carbonate solution (8%, *w*/*v*) in water (0.4 mL) after 5 min and DI was added to make the volume to 1.2 mL. Absorbance was measured at 765 nm after incubating the mixture for 30 min at RT. TPC was expressed in terms of mg Gallic acid equivalents (GAE)/g of dry plant material [38]. Aluminum chloride colorimetric assay was used to determine the total flavonoid content (TFC). A portion of about 0.5 mL extracts as sample and quercetin as a standard solution (5–250 μg/mL) and 0.5 mL of 2% AlCl_3_ solution were mixed, incubated for 60 min at RT. Absorbance was measured at 430 nm, and the TFC was expressed in terms of mg quercetin equivalents (QE)/g of dry plant material [38].

#### 2.5.2. DPPH Free Radical Scavenging Assay

The DPPH free radical scavenging assay was completed by mixing BG-AgNPs (125–500 µg/mL), and ascorbic acid used as standard control solution with 0.04% (*w*/*v*) DPPH suspended in ethanol of about 200 µL each kept in the dark for 30 min at room temperature [39]. The absorbance was measured at 521 nm against a blank of 0.02% DPPH after incubating in the dark for 30 min at RT. The DPPH free radical scavenging activity was expressed as follows:

DPPH scavenging effect (%) = [(A_0_ − A_t_)/A_0_] × 100

A_0_ is the absorbance of the control and at is the absorbance of the sample.

#### 2.5.3. Ferric Reducing Antioxidant Power (FRAP) Assay

FRAP assays of the bitter gourd synthesized AgNPs (25–100 µg/mL) in water were evaluated by the reduction of ferric 2,4,6-tripyridyl-s-triazine complex (Fe^3+^-TPTZ) to colored ferrous form (Fe^2+^-TPTZ) with some modification in the reported literature. FRAP reagent was prepared by mixing 2.5 mL of TPTZ solution (10 mM TPTZ in 40 mM HCl) and FeCl_3_ (20 mM) in 25 mL of acetate buffer (0.3 M, pH 3.6). The freshly prepared 650 µL of FRAP reagent was mixed with 75 µL of respective BG-AgNPs. In addition, ascorbic acid acted as a standard control, whereas DI water was used as the reagent blank. Absorbance at 593 nm was measured after incubating the reaction mixture at 37 °C for 30 min [39].

### 2.6. Antibacterial Activity of BG-AgNPs

#### 2.6.1. Dissolution of Ag Ions from BG-AgNPs

In total, 200 ppm of A-BG- and E-BG-AgNPs were first dispersed in DI water, respectively. An aliquot of the BG-AgNP solution (5 mL) was harvested after 0.5, 1, 3, 6, and 24 h of aging periods. After centrifugation at 15,000× *g* for 20 min, the resulting supernatant solutions were collected and diluted with 5 M nitric acid for the measurement of Ag concentrations using atomic absorption spectrophotometry (AAS) [40].

#### 2.6.2. Minimum Inhibitory Concentration (MIC)

The minimum inhibition concentration (MIC) was performed using the broth dilution method in three repeated sets of experiments against different bacterial strains such as *E. coli*, *P. aeruginosa*, *A. baumannii*, *S. aureus*, as well as colistin- and imipenem-resistant *A. baumannii*. In a 96-well microplate, A-BGE, E-BGE, and the respective BG-AgNPs of various concentrations (1–64 µg/mL) were mixed with 10^6^ CFU/mL of respective bacterial strains in Mueller-Hinton broth and incubated at 37 °C for 18 h. The MIC value was measured by measuring OD at 600 nm or no visible growth of bacterial strains [41]. Chemically synthesized AgNPs (14 nm) using NaBH_4_ was used as blank control according to the previous report [42].

#### 2.6.3. Instant Antibacterial Activity

BG-AgNPs (8 µg/mL) was mixed with 10^6^ CFU/mL of *E. coli* at 37 °C for 2.5 h in a shaking incubator. The mixtures were diluted to10^4^ CFU/mL before spreading uniformly onto Miller-Hinton agar plates [43]. The survival rate (%) of bacterial cells was calculated by counting the CFU.

#### 2.6.4. Time-Dynamic Antibacterial Test

A time-dynamic antibacterial test was performed to evaluate the antimicrobial activity of A-BG-AgNPs at different incubation times. *E. coli* and *S. aureus* cells (10^7^ CFU/mL) were, respectively, incubated with A-BG-AgNPs (8 and 16 μg/mL for *E. coli* and *S. aureus*) in PBS buffer. A bacterial control group for *E. coli* and *S. aureus* was incubated in the absence of A-BG-AgNPs. Bacteria were sampled from 0 to 4 h, diluted, and cultured on LB plates to estimate the survival rate (%) at each time point.

#### 2.6.5. Reactive Oxygen Species (ROS) Measurement

ROS measurement was performed by mixing 5 × 10^7^ CFU/mL of *E. coli* and 80 mg/mL of A- and B-BG-AgNPs for 2 h, respectively. After washing with phosphate-buffered saline (PBS) two times, the mixtures were mixed with 25 µM fluorescent dye 2′,7′-dichlorofluorescein-diacetate (DCFH-DA) for 30 min. The DCFH-DA-labeled bacteria were further washed twice with PBS before recording the fluorescence images. Olympus IX71 microscope (Tokyo, Japan) with a SPOT RT3 digital camera was used to capture the microscopic images with the excitation at 510–530 nm [41].

#### 2.6.6. Electron Microscopy Experiments

BG-mediated AgNPs were incubated for 30 min at 37 °C with *E. coli*, *S. aureus*, and *A.* *baumannii* (10^9^ CFU) in Mueller–Hinton medium, respectively. Cell pellets were fixed with glutaraldehyde (2.5%, *w*/*v*) in 0.1 M cacodylate buffer and 1% tannic acid for 1 h at 4 °C. The cells were washed with DI and PBS twice, followed by dehydrating with ethanol. The Control group of *E. coli* cells was treated with PBS (pH 7.4) in this study. After critical-point drying and gold coating, the cells were examined under a scanning electron microscope (S-4700; Hitachi, Tokyo, Japan). A drop containing the bacteria was deposited onto a carbon-coated grid with 2% uranyl acetate, and the grids were examined using a transmission electron microscope (H-7500; Hitachi) [41].

#### 2.6.7. In Vitro Hemolysis Assay

The hemolytic activity against human red blood cells (RBCs) by BG mediated AgNPs was evaluated using a published protocol with some modification [44]. RBCs were freshly harvested and centrifuged for 10 min at 4 °C at the speed of 3500 *g* and washed three times with 10 mM PBS buffer. Then, 50 μL of various concentrations (31.25–4000 μg/mL) BG-AgNPs were incubated with 500 μL of 4% erythrocyte suspension at 37 °C for 1 h. The mixture was centrifuged at 3500 *g* for 10 min, and the obtained supernatant of about 200 μL was transferred into the 96-well plate to measure the OD at 600 nm. RBCs with 10 mM PBS and 0.1% (*v*/*v*) Triton X-100 solution were used as control to determine the 0% and 100% lysis efficiency, respectively [41].

### 2.7. Statistical Analysis

All the experiments in the present investigation were performed in triplicates individually to ensure reproducibility. Microsoft Excel 2016 was used to calculate the mean and standard deviations. The graphs, bar charts were prepared using Origin Pro 8.5 software (Northampton, MA, USA).

## 3. Results

### 3.1. Characterization of Biosynthesized Silver Nanoparticles Using the BG Extract

**Scheme 1:**
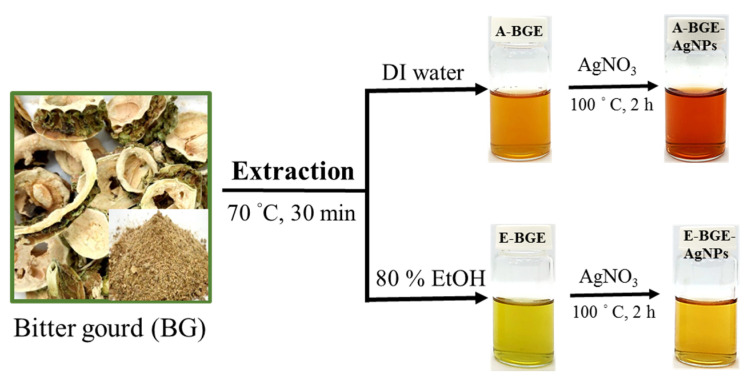
Preparation of BG extracts using deionized water and 80% ethanol to synthesize BGE mediated AgNPs.

The formation and optical properties of obtained BG-mediated AgNPs were confirmed by visual observation and measuring their surface plasma resonance (SPR) band through UV-vis absorption spectroscopy. We chose deionized water (DI) and 80% ethanol as eco-viable green solvents for the preparation of the aqueous and ethanolic BG extracts (i.e., A-BGE and E-BGE, Scheme 1). While incubating AgNO_3_ with A-BGE and E-BGE at 100 °C for 2 h, we found that a reddish-brown color of A-BG-AgNPs was synthesized using the A-BGE as a reducing and stabilizing reagent, whereas the ethanolic counterpart changed its color from green to yellowish-brown for the generation of E-BG-AgNPs.

**Figure 1:**
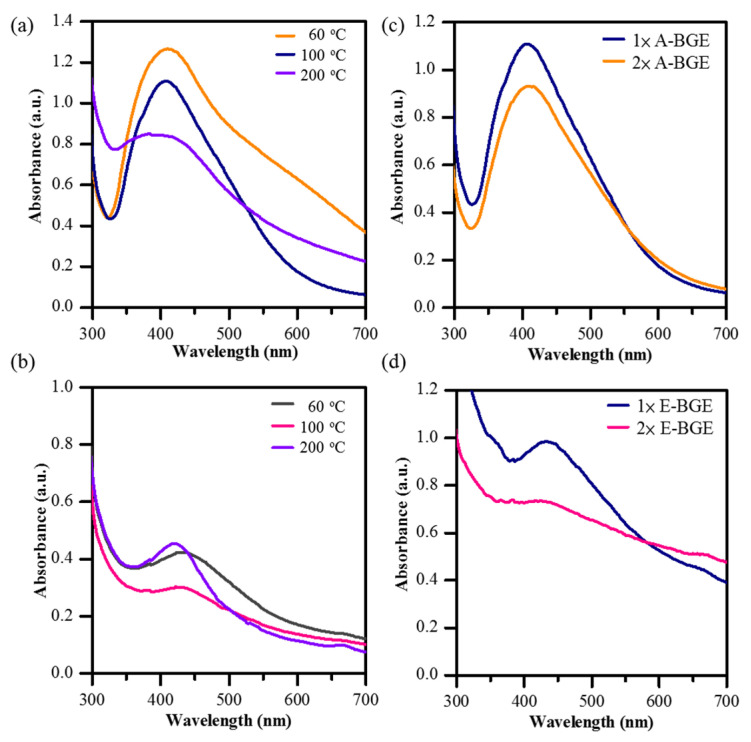
UV–vis spectra of purified A-BG-AgNPs and E-BG-AgNPs prepared at different (**a**,**b**) reaction temperatures and (**c**,**d**) BGE concentrations.

As shown in Figure 1, the purified AgNPs showed an SPR absorbance peak at 408 nm for A-BG-AgNPs and 422 nm for E-BG-AgNPs, respectively. The A-BG-AgNPs exhibited a much sharper and blue shift SPR peak, whereas the SPR band for E-BG-AgNPs was broader and had a red shift. We found that purified A-BG-AgNPs were stable even after a 2-week storage duration, whereas E-BG-AgNPs showed aggregation within 1 h after purification.

The results of the UV-vis absorption measurement (Figure 1) display the effect of temperature and CGE proportion for the generation of BG-AgNPs. As shown in Figure 1a, UV-vis spectra of A-BG-AgNPs synthesized at 60, 100, and 200 °C showed a respective, maximum absorbance at 410, 408, and 384 nm, whereas the red-shift of the SPR bands for E-BG-AgNPs at 430, 422, and 418 nm were observed (Figure 1b), respectively. We then prepared A-BGE and E-BGE from two different BG material proportions (i.e., 1 and 2 g in 50 mL corresponding solvent, named as 1 and 2× BGE). Figure 1c,d reveals that the A-BG-AgNPs possessed SPR bands at 408 and 410 nm using 1 and 2× A-BGE, whereas the maximum absorbance of E-BG-AgNPs was prepared by 1 and 2× E-BGE were at 432 and 424 nm, respectively.

**Figure 2:**
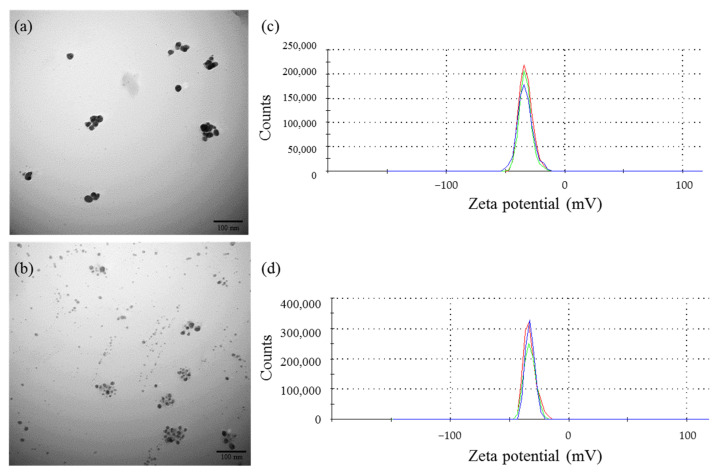
TEM images (**a**) A-BG-AgNPs and (**b**) E-BG-AgNPs. Zeta potential values (*n* = 3) of (**c**) A-BG-AgNPs and (**d**) E-BG-AgNPs.

A TEM micrograph confirmed the average size and morphology of the AgNPs. As shown in Figure 2a,b, the average sizes of A-BG- and E-BG-AgNPs were 16.4 ± 4.9 and 9.6 ± 3.7 nm, respectively. Both AgNPs were monodispersed and spherical. However, the ethanolic AgNPs were much smaller in size and showed greater agglomeration than the aqueous ones. The surface charge on the AgNPs in aqueous media was determined by measuring the Zeta potential (Figure 2c,d). Negative Zeta potential values of −33.0 ± 0.6 and −32.7 ± 0.5 mV were observed (*n* = 3) for the A-BG-AgNPs and E-BG-AgNPs, respectively.

**Figure 3:**
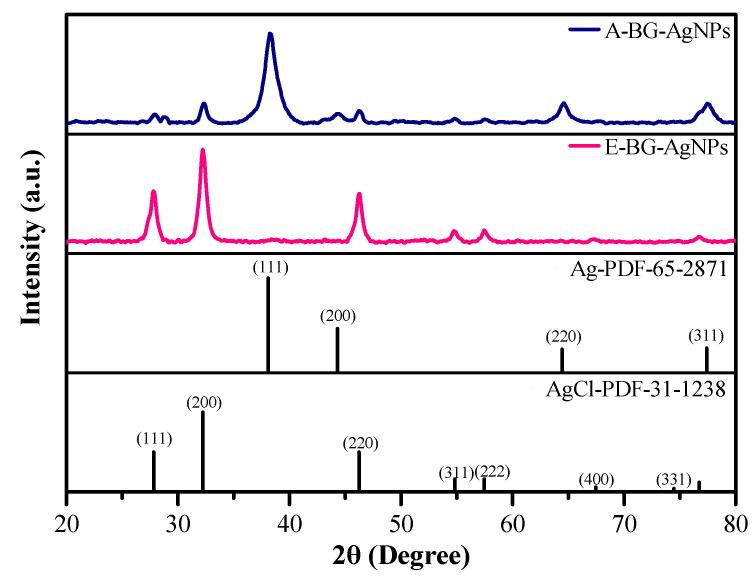
X-ray diffraction pattern of A- and E-BG-AgNPs.

The crystalline structures of A-BGE and E-BGE synthesized AgNPs were elucidated by the XRD diffraction patterns. As shown in Figure 3, the diffraction peaks of the AgNPs at 27.8°, 32.3°, 46.2°, 54.9°, and 57.7° were indexed to the (111), (200), (220), (311), and (222) reflection plane corresponding to the AgCl (PDF-31-1238), respectively, due to the crystallization of the bio-organic phase in the extract for both A-BG-AgNPs and E-BG-AgNPs [45]. Whereas, some extra diffraction peaks were observed only for A-BG-AgNPs at 38.3°, 44.2°, 64.6°, and 77.5°, which were indexed to the (111), (200), (220), (311) crystal planes of Ag (PDF-65-2871). This confirms the presence of Ag and AgCl in the A-BG-AgNPs, whereas E-BG-AgNPs possessed AgCl on the AgNPs’ surface.

**Figure 4:**
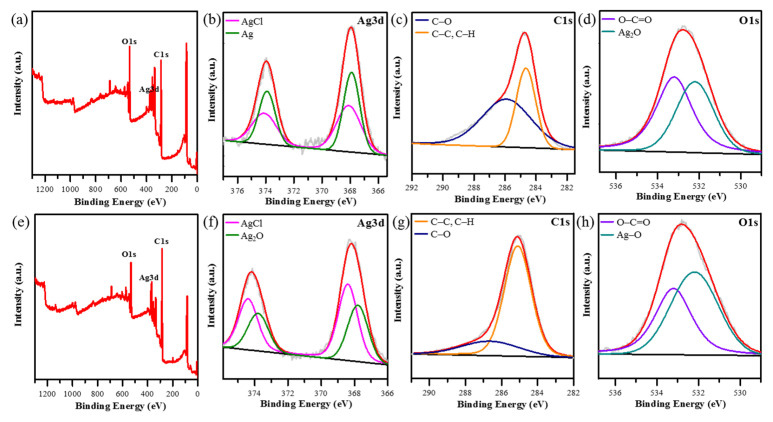
XPS spectra of the (**a**,**e**) survey, (**b**,**f**) Ag 3d, (**c**,**g**) C1s, and (**d**,**h**) O1s of A-the BG-AgNPs and E-BG-AgNPs, respectively.

The elemental composition and oxidation state of BG-mediated AgNPs were studied using X-ray photoelectron spectroscopy (XPS). The full-scan spectrum of A-BG-AgNPs and E-BG-AgNPs, along with the analysis of the detailed elements of carbon (C1s), oxygen (O1s), and silver (Ag3d), are presented in Figure 4. In Figure 4b, the high-resolution Ag3d spectrum of A-BG-AgNPs corresponds to Ag3d_3/2_ and Ag3d_5/2_, with binding energies (BEs) at 368.1 and 374.1 eV. Both signals can be further fitted to several symmetrical peaks with BEs at 367.9 and 368.1 eV, which correspond to metallic Ag and AgCl, respectively. For the XPS analysis of C1s and O1s signals, Figure 4c reveals these peaks can be deconvoluted into C–O (285.9 eV), and C–H, or C–C (284.6 eV), whereas peaks from the O1s data (Figure 4d) are assignable to AgO (532.2 eV) and O–C=O (533.2 eV). For the E-BG-AgNPs, all of the elemental compositions were similar to that of the A-BG-AgNPs, except for the Ag element. The Ag3d spectrum of the E-BG-AgNPs (Figure 4f) showed deconvoluted peaks at 367.8 and 368.1 eV, belonging to AgCl and Ag_2_O, respectively [46].

**Figure 5:**
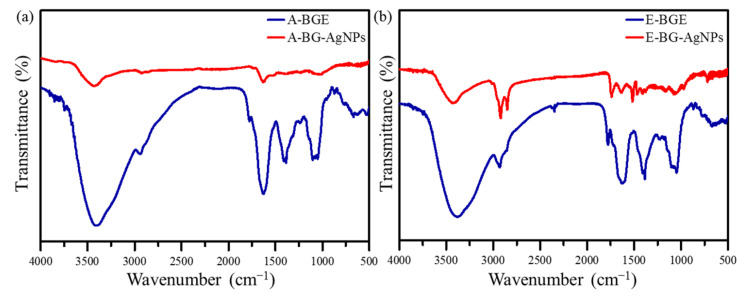
FT-IR spectra of (**a**) A-BGE and A-BG-AgNPs; (**b**) E-BGE and E-BG-AgNPs.

In Figure 5a,b, the FT-IR spectra of A-BGE and E-BGE showed similar peak patterns at approximately 3425, 2943, 1637, 1386, and 1106 cm^–1^, respectively, corresponding to O–H stretching (alcohol), C–H stretching (alkane), N–H bending (amines) or C=C stretching (alkene), C–H bending (aldehyde), and C–O stretching (primary alcohol) or C–N stretching (amine). In addition, E-BGE also displayed all of the peaks mentioned above, with an extra sharp peak at approximately 1780 cm^–1^, detected and assigned to C=O stretching of conjugated acid halide. The functional groups present in bitter gourd extracts are in agreement with previous studies [28,29]. FT-IR data also pointed out that both the BG-AgNPs showed peak shifts with a decrease in peak intensities at approximately 3430, 2929, and 1631 cm^–1^, as well as the disappearance of a few characterized peaks. The E-BG-AgNPs showed an extra intense peak at 2851 cm^–1^, corresponding to N–H stretching of the amine group.

**Figure 6:**
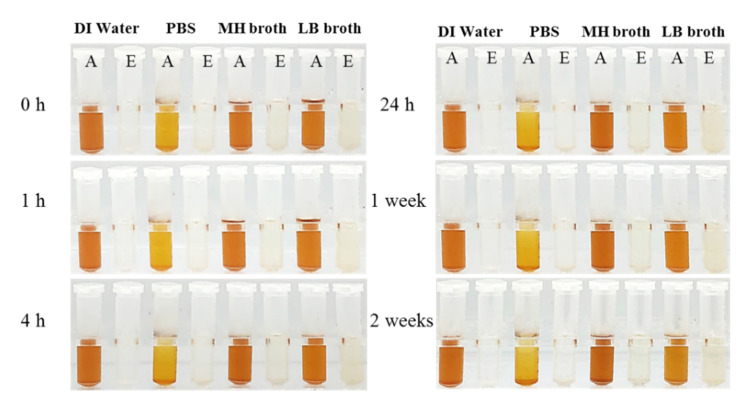
Images of (A) A-BG-AgNPs and (E) E-BG-AgNPs (50 μg/mL) in different media after incubation at the indicated time.

To determine the long-term stability of Ag nanomaterials in various biological media, A- and E-BG-AgNPs were dispersed in DI water, phosphate-buffered saline (PBS), Mueller-Hinton (MH) broth, and Luria-Bertani (LB) broth for different intervals of time [43]. As shown in Figure 6, the A-BG-AgNPs were stable in aqueous-based media for 2 weeks, whereas the E-BG-AgNPs completely precipitated after 1 h. These results indicate that A-BG-AgNPs exhibit higher degrees of stability in physiological solutions.

### 3.2. Quantification of Phytoconstituents and In Vitro Antioxidant Activity

**Figure 7:**
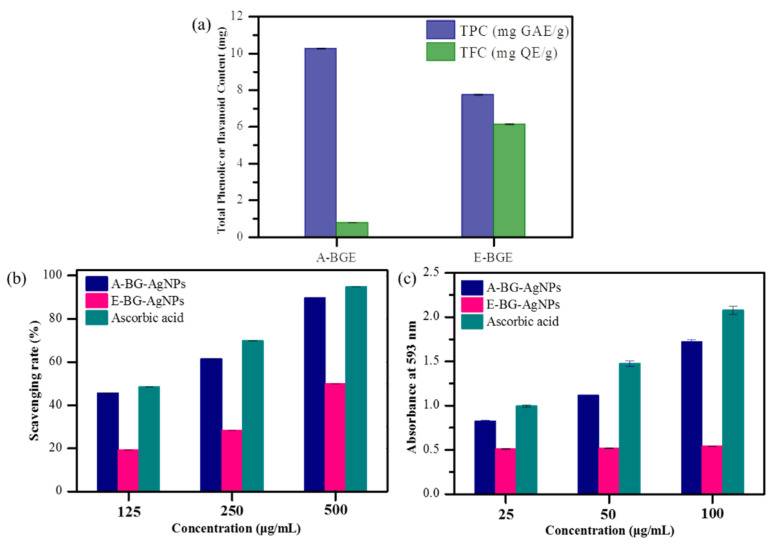
(**a**) Total phenolic and flavonoid content analysis of the BG extracts. (**b**) DPPH radical scavenging activity and (**c**) ferric-reducing ability assays of the BG-AgNPs and ascorbic acid. Each test was performed in triplicate and repeated three times individually.

The TPC and TFC assays were conducted to determine the total phenolic and flavonoid content present in the BG extracts. While using gallic acid and quercetin as the reference [47], Figure 7a indicates that both the aqueous and ethanolic BG extracts possessed different degrees of phenolic and flavonoid contents. We noticed that A-BGE had a higher phenolic content of 10.2 mg GAE/g compared to E-BGE (7.7 GAE/g). Interestingly, the TFC results showed less flavonoid content in A-BGE (0.7 mg QE/g) than in E-BGE (6.1 mg QE/g).

To further understand the antioxidant performance of BG-AgNPs, 2,2-diphenyl-1-picryl-hydrazyl-hydrate (DPPH) radical scavenging activity and the ferric-reducing ability assays were also examined in this study. Figure 7b reveals that both the BG-mediated AgNPs showed increases in the scavenging rate with an increase in the doses of AgNPs. In addition, the A-BG-AgNPs provided the best scavenging rate of 45.6%, 61.3%, and 89.8% at 125, 250, and 500 µg/mL of AgNP concentration, whereas E-BG-AgNPs displayed weaker radical scavenging efficiency (i.e., 19.1%, 28.1%, and 49.7%, respectively). The obtained results were compared with ascorbic acid as a control. The ferric reducing antioxidant power (FRAP) activity of different BG-AgNPs was performed by measuring the reducing potential of antioxidants on reaction with a colorless ferric tripyridyltriazine [Fe^3+^-TPTZ] complex to produce blue-colored ferrous tripyridyltriazine [Fe^2+^-TPTZ]. Since the increase in absorbance at 593 nm was proportional to the reducing ability of the tested sample [47], ascorbic acid was used as a standard solution in this assay. Figure 7c shows that the A-BG-AgNPs provided a better reducing power of antioxidants than the E-BG-AgNPs at different tested nanoparticle concentrations.

### 3.3. Concentration of Ag Ions Released from BG-AgNPs

**Figure 8:**
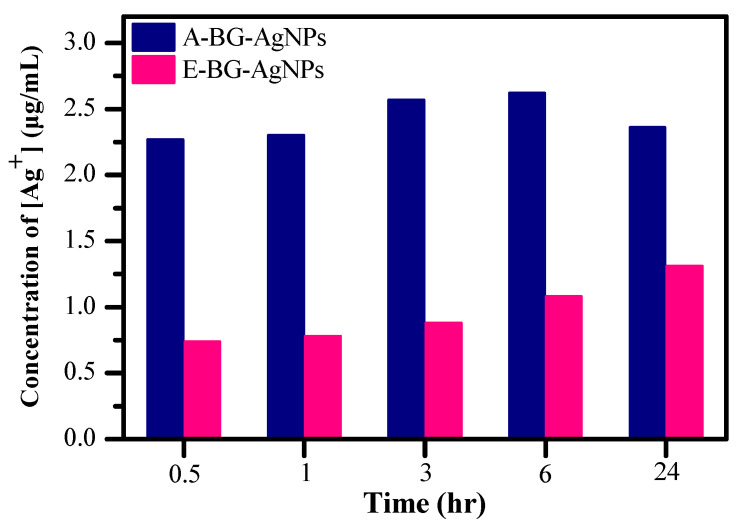
AAS measurements for the release of Ag ions from A-BG-AgNPs and E-BG-AgNPs at different incubation times, respectively.

We performed the AAS measurements to calculate the concentration of silver ions released from BG-mediated AgNPs. As shown in Figure 8, the concentration of Ag^+^ released from A-BG-AgNPs (200 ppm) was 2.27, 2.30, 2.57, 2.62, and 2.36 ppm, whereas 0.74, 0.78, 0.88, 1.08, 1.31 ppm of Ag ions were released from E-BG-AgNPs (200 ppm) at 0.5, 1, 3, 6, and 24 h, respectively. Thus, the concentration of released Ag^+^ ions from A-BG-AgNPs was found to be around 2-fold greater than the E-BG-AgNPs at different incubation times.

### 3.4. Antibacterial Efficiency and Biocompatibility of Biosynthesized AgNPs

BG-mediated AgNPs were evaluated for antibacterial activity by assessing the minimum inhibition concentration (MIC) experiments against *Escherichia coli*, *Pseudomonas aeruginosa*, *Acinetobacter baumannii* (wild type), *Staphylococcus aureus* (non-MDR strains), and two drug-resistant Gram-negative strains, namely, colistin- (CR32X 17978) and imipenem-resistant (IMP32X 17978) *A. baumannii*. As shown in Table 1, both aqueous and ethanolic BG extracts mediated AgNPs showed similar antibacterial effectiveness against the *E. coli*, *A. baumannii*, CR32X17978, and IMP32X17978 strains, with a MIC value of 4 μg/mL. It seems that the A-BG-AgNPs possessed superior antibacterial efficacy compared to the E-BG-AgNPs, which had MIC values of 2 µg/mL against *P. aeruginosa* and 4 µg/mL against *S. aureus*, respectively. In addition, the MIC value of A-BG extract, E-BG extract, and AgNPs synthesized using NaBH_4_ [42] was greater than 64 µg/mL against all tested bacterial strains, respectively.

The instant antibacterial activity was carried out by counting the CFUs of *E. coli* strains after reaction with the respective A- and E-BG-AgNPs. The incubation procedure was performed by mixing BG-AgNPs with *E. coli* at 37 °C for 2.5 h, and an aliquot of the suspension was added to the MH agar plate. The survival rate (%) of *E. coli* was determined through the CFU counting procedure. While incubating with 8 µg/mL of BG-AgNPs, 95.2% and 89.4% of *E. coli* were killed using A-BG-AgNPs and E-BG-AgNPs, respectively. This indicates that A-BG-AgNPs displayed a stronger instant antimicrobial activity against *E. coli* than the BG-AgNPs.

**Figure 9:**
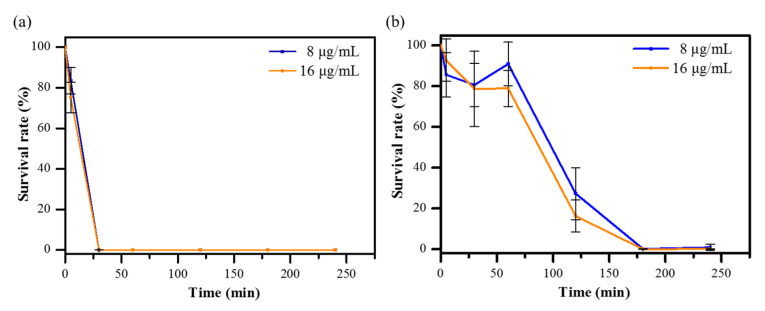
Time dynamic survival rate curves of (**a**) *E. coli.* and (**b**) *S. aureus* treated with different concentrations of A-BG-AgNPs (*n* = 3).

Due to the A-BG-AgNPs showing better antibacterial performance than E-BG-AgNPs, we further investigated the time-dynamic antibacterial activity of A-BG-AgNPs towards *E. coli* and *S. aureus*. As shown in Figure 9, the survival rates of the tested bacteria are dependent on the concentration of the A-BG-AgNPs and exposure time. After treating with 8 and 16 μg/mL A-BG-AgNPs for 5 min, Figure 9a shows that the survival rate of *E. coli* decreased to 84% and 75%, whereas 0% of bacterial cells survived after 30 min. While *S. aureus* was exposed to A-BG-AgNPs, Figure 9b reveals that the survival rate was down to 81% and 79% after 30 min incubation with 8 and 16 μg/mL A-BG-AgNPs, respectively. The survival curve of *S.aurues* was gradually declined to 0% after 3 h incubation.

**Figure 10:**
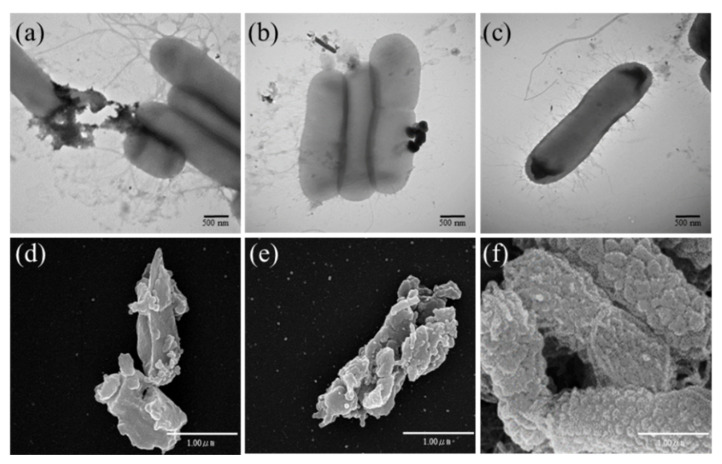
TEM (**a**–**c**) and SEM (**d**–**f**) images of *E. coli* with and without treatment with AgNPs (20 µg/mL). The *E. coli* cells were treated with (**a**,**d**) A-BG-AgNPs and (**b**,**e**) E-BG-AgNPs, and (**c**,**f**) show untreated bacteria cells.

Electron microscopy experiments were performed to determine the physiochemical changes occurring in the bacterial cells while interacting with the BG-mediated silver nanoparticles. All these morphological changes were compared with the untreated *E. coli* cells as a control. The TEM images in Figure 10a,b reveal that both BG-AgNPs could adhere to the surface of the *E. coli* cells, whereas the interruption of bacterial membranes/walls was significantly induced after exposure to A-BG-AgNPs. In addition, SEM measurements (Figure 10d–f) further confirm that the *E. coli* cells were treated with BG-AgNPs leading to severe damage to the cell structure [48,49].

**Figure 11:**
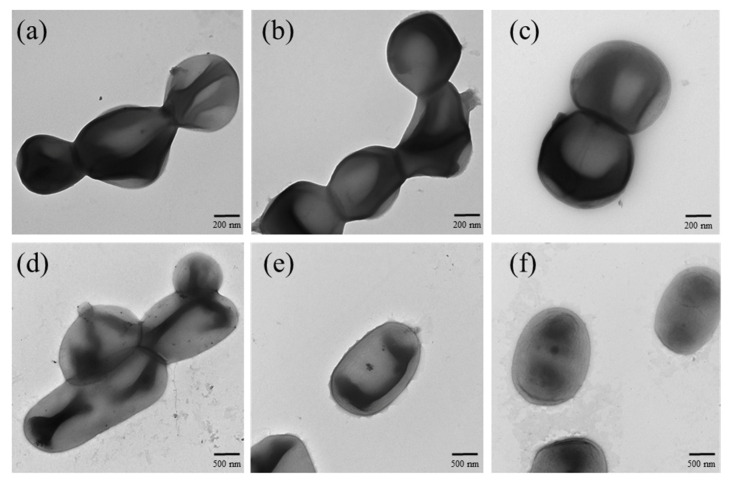
TEM images of *S. aureus* treated with (**a**) A-BG-AgNPs, (**b**) E-BG-AgNPs and (**c**) untreated bacteria cells, respectively. TEM images of *A. baumannii* treated with (**d**) A-BG-AgNPs, (**e**) E-BG-AgNPs, and (**f**) untreated *A. baumannii* cells, respectively.

We further performed the TEM measurements to evaluate the structural changes of *S. aureus* and *A. baumannii* while adding BG-AgNPs. While *S. aureus* was incubating with BG-AgNPs (32 µg/mL), bacterial cells failed to retain their original shapes (Figure 11a,b) compared with the untreated *S. aureus* cells (Figure 11c). However, in the case of *A. baumannii*, Figure 11d,e reveals that the BG-AgNPs (32 µg/mL) could adhere to the surface of *A. baumannii*, leading to a mild change in the morphology of cell when compared with the control group (Figure 11f).

**Figure 12:**
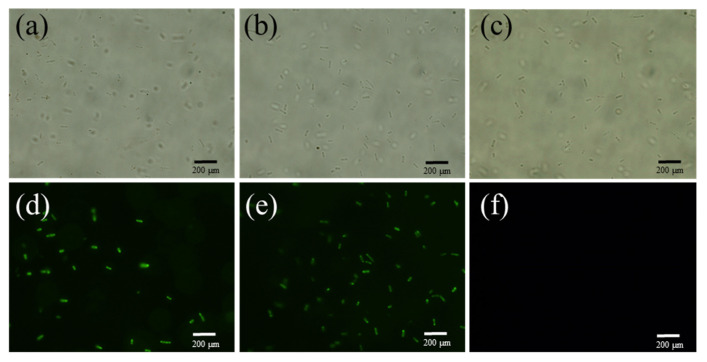
(**a**–**c**) Bright-field and (**d**–**f**) fluorescence images of *E. coli* incubated with (**a**,**b**) A-BG-AgNPs and (**b**,**e**) E-BG-AgNPs, and (**c**,**f**) show untreated bacteria after DCFH-DA treatment.

We evaluated the ability of BG-mediated AgNPs to produce ROS by using the 2′,7′-dichlorodihydrofluorescein diacetate (DCFH-DA) assay. This probe is deacetylated by esterase to form non-fluorescent DCFH that reacts with ROS to form green fluorescent 2′,7′-dichlorodihydrofluorescein (DCF) that is trapped inside the cell, making the cell fluorescent. Compared to the untreated *E. coli* control (Figure 12f), we observed that A-BG-AgNP (Figure 12d) and E-BG-AgNP (Figure 12e) treated *E. coli* induced the generation of higher ROS levels.

**Figure 13:**
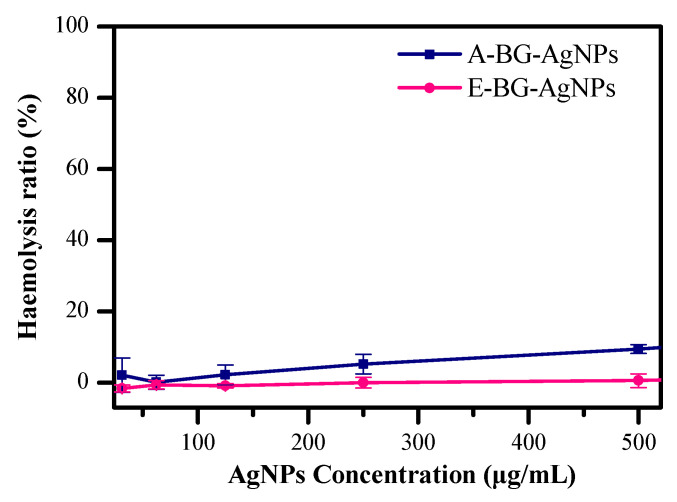
Hemolytic activities of BG-mediated AgNPs against human RBCs (*n* = 3).

Hemolytic evaluations were performed to determine the biocompatibility of BG extract stabilized AgNPs by incubating human red blood cells (RBCs) with different concentrations (31.25–500 μg/mL) of BG-AgNPs. Figure 13 reveals that the viabilities of the RBCs were 90.6% in the presence of 500 μg/mL of A-BG-AgNPs, which is 125- and 250-fold higher than the MIC to tested drug-resistant Gram-negative bacteria strains and *P. aeruginosa*. There was no visible cell damage detected by adding the same amount of E-BG-AgNPs. It was observed that BG-AgNPs showed <10% hemolytic activity even with a higher concentration [50]. Collectively, our results reveal that the A-BG-AgNP is a compromising antioxidant and antimicrobial nanomaterial, which has high-dose safety for the treatment of mammalian cells.

## 4. Discussion

This study investigated the potential antioxidant efficacy and possible antibacterial activity of green synthesis of AgNPs using bitter gourd extract. While using deionized water and ethanol as a solvent for the preparation of BGE, these two solvents have been shown to provide efficient extraction of heterogeneous phytochemicals and less solvent-related environmental damage [51]. Among all the tested biosynthesis parameters and the preliminary experiments performed, we found that the purified A-BG-AgNPs, using 1× of A-BGE and incubated at 100 °C for 2 h, showed a narrower SPR peak and 2 weeks of dispersed stability compared to the E-BG-AgNPs (i.e., 10 min) in deionized water. Furthermore, TEM images revealed that the average size of the A-BG-AgNPs was larger than for the E-BG-AgNPs. In contrast, both BG-AgNPs possessed high negative zeta potential values, which makes the nanosuspensions highly stable and biocompatible. The negative zeta potential values of the BG-AgNPs are due to the several negatively charged phytochemicals surrounding AgNPs’ surface [6,52].

We noticed that our XPS studies on BG-AgNPs were highly correlated with the XRD results, which shows that A-BG-AgNPs have more metallic Ag along with minor AgCl on the Ag surface, whereas the E-BG-AgNPs possessed AgCl and Ag_2_O. Our observations are similar to those reported by Okaiyeto et al. and Devi et al., showing that the formation of AgCl might be due to the reaction between Ag^+^ of AgNO_3_ and the Cl^–^ from the phytochemicals in the BG extracts. Upon the AgCl formation, the Ag^+^ was also reduced to metallic Ag due to the reducing ability of phytochemicals present in the BG extracts. Later, Ag^+^ forms an intermediate complex with the phenolic compounds present in the plant extract by undergoing oxidation and reducing Ag^+^ to Ag^0^ [53,54]. Compared to corresponding BGE, our FT-IR results indicate that several intense FT-IR peaks belonging to phytochemicals in BGE decreased in intensity or disappeared for the BG-AgNPs. We speculate that the biomolecules present in the extracts were utilized in the bio-reduction of Ag^+^ to AgNPs and capped on the surface of AgNPs [55].

To further evaluate the possible antioxidant activity of A- and E-BG-AgNPs, we first aimed to understand the total phenolic and flavonoid contents of as-prepared BG extracts. Our TPC and TFC results indicated that the major constituents in the aqueous BGE were phenolic compounds [56]. In contrast, the E-BGE was composed of similar composition of phenolic compounds and flavonoids. Collectively, we concluded that the aqueous bitter gourd extracts exhibited higher phenolic content. Therefore, it can be responsible for reducing Ag ions into AgNPs and acting as the capping/stabilizing agent on AgNPs’ surface.

According to the results from the DPPH and FRAP assays, the antioxidant activity of A-BG-AgNPs seems to provide a more vital antioxidant efficiency than E-BG-AgNPs within a dose-dependent manner. It has been reported that cucurbitane-type triterpenoids, phytosterols, carotenoids, protein, phenolic compounds, fatty acids, essential oils, carotenoids, saponin, and alkaloids, are considered as the main bioactive compounds present in the seeds and fruits of bitter gourd [19,47]. We believe that the potential antioxidant ability of A-BG-AgNPs is mainly due to the phenolic constituents present in the extracts. Phenolic compounds possess several antioxidant properties, such as donating hydrogen atoms, breaking the free radical production cycle to interrupt the chain reactions, and preventing oxidative stress [57]. It has been reported that the AgNPs synthesized using plant extracts exhibit higher antioxidant and scavenging ability due to the phenolics and flavonoids that existed in the extracts [58].

In general, the stability of nanomaterials in different physiological media is highly correlated with their antimicrobial effectiveness [43]. Our stability test of nanomaterials displays that A-BG-AgNPs remain to disperse stably over two weeks after storage in various aqueous solutions than E-BG-AgNPs. The instability of E-BG-AgNPs is due to the presence of Cl ions in the plant extract leading to the formation of AgCl, further hindering the synthesis of AgNPs and affecting their long-term stability. This is in agreement with the studies from Siakavella et al. [59] and Li et al. [60].

The results of the MIC tests for BG-AgNPs, BG extracts, and NaBH_4_ synthesized AgNPs, against Gram-negative, Gram-positive, and drug-resistant Gram-negative bacterial strains also indicated that the A-BG-AgNPs provided stronger antibacterial efficiency against all tested bacteria. In general, metal nanoparticles synthesized under conventional chemical methods exhibit limited antimicrobial activities. In contrast, the combination of plant extracts and AgNPs induces the synergistic effect to enhance the antimicrobial effectiveness of plant extract-mediated AgNPs [61]. As shown in Table 2, A-BG-AgNPs showed a lower MIC value than other green synthesized AgNPs against *E. coli* and *S. aureus*.

Our AAS measurements revealed that 2-fold more of Ag^+^ ions were released from A-BG-AgNPs than those of E-BG-AgNPs in aqueous media. Tang et al. [71] speculated that antibacterial activities of AgNPs are also related to silver ion release, which could be generated during the oxidative dissolution of AgNPs in the presence of oxygen. Thus, the released Ag ions from the BG-AgNPs might have effectively taken part in the antibacterial activity as the silver ions possess a high affinity to electron-donating groups present on the bacterial cell membranes. In addition, the instant antibacterial capacity test showed that A-BG-AgNPs inhibited 95.2% of *E. coli* from survival. After exposure to A-BG-AgNPs for 30 min, the decrease in the survival rate of *E. coli* gradually stabilized to 0%. In contrast, no live *S. aureus* were detected after 3 h incubation with A-BG-AgNPs. Based on our findings, we propose that 30 min and 3 h is a reasonable treatment duration for A-BG-AgNPs against *E. coli* and *S. aureus*, respectively. Zhao et al. [43] reported that the differences in the curves between *E. coli* and *S. aureus* might be due to their different cell wall structure and composition. As AgNPs can easily destroy the thin outer structure of *E. coli* leading to apoptosis, while the thick and dense peptidoglycan layer in the cell wall of *S. aureus* provides delay apoptosis. Moreover, hemolytic evaluations of BG-AgNPs against human RBCs demonstrated that the BG-mediated AgNPs were highly biocompatible to mammalian cells. To our best knowledge, the inhibition efficiency of A-BG-AgNPs against drug-resistant Gram-negative bacterial strains is superior to the other plant extract-mediated AgNPs from previous studies [72,73].

To investigate the interaction between Ag nanomaterials and bacteria, our EM experiment points out that the surface structure of *E. coli* became rough and damaged after exposure to BG-AgNPs, whereas the significant number of *S. aureus* and *A. baumannii* cells also showed the morphological changes in the addition of BG-AgNPs. While silver nanoparticles were interacting with microorganisms, various reactive oxygen species (ROS), such as hydrogen peroxide (H_2_O_2_), hydroxyl radicals (•OH), superoxide anion radicals (O_2_•), and per hydroxyl radicals (HOO•), were reported to be generated [74]. Compared to the untreated *E. coli* cells, our DCFH-DA assay further demonstrated that a higher level of ROS was generated while BG-AgNPs interacted with *E. coli*. In summary, the antibacterial mechanism of BG-AgNPs is mainly attributed to the release of Ag and Ag^+^ from the AgNPs’ surface [75], for the ROS production and physical damage to the bacterial structure [41]. As reported by vanlalveni et al. [12], although the usage of plant extracts has shown great potential in the synthesis of AgNPs, understanding the mechanism by which phytochemicals of these plants are responsible for the antimicrobial inhibition is still being explored.

## 5. Conclusions

Herein, we provided a green synthesis route for the preparation of AgNPs with efficient antioxidant and antibacterial properties using different bitter gourd extracts. Compared to the E-BG-AgNPs, the A-BG-AgNPs possessed a much sharper and blue shift SPR peak at 408 nm, without apparent aggregation in various aqueous media. Both XRD and XPS surface characterizations indicated the presence of metallic Ag and AgCl on the A-BG-AgNPs’ surface. While estimating the free radical scavenging rate for DPPH and the antioxidant ability for the BG-AgNPs, the A-BG-AgNPs displayed a higher degree of antioxidant performance due to the greater abundance of phenolic compounds that existed in the aqueous BG extracts and AgNPs. The MIC tests revealed that both BG-AgNPs showed limited hemolysis of RBCs and excellent antibacterial activity against non- and drug-resistant bacterial strains. Our EM studies further support that the bacterial membrane/wall damage through ROS production is one of the possible antibacterial mechanisms while BG-AgNPs interact with pathogens.

## Figures and Tables

**Table 1:** Minimum inhibition concentration (µg/mL) of different Ag NPs and BG extracts.

Pathogenic Bacteria	A-BG-AgNPs	E-BG-AgNPs	A-BG Extract	E-BG Extract	AgNPs Synthesized Using NaBH_4_
*E. coli*	4	4	>64	>64	>64
*P. aeruginosa*	2	2	>64	>64	>64
*A. baumannii*	4	4	>64	>64	>64
*S. aureus*	4	16	>64	>64	>64
Colistin-resistant *A. baumannii*	4	4	>64	>64	>64
Imipenem-resistant *A. baumannii*	4	4	>64	>64	>64

**Table 2:** Comparison of MIC against *E. coli*, and *S. aureus* by AgNPs synthesized using different plant extracts.

Plant Extract Mediated AgNPs	Size (nm)	MIC (μg/mL) ^a^	MIC (μg/mL) ^b^	Reference
*Alpinia katsumadai*	12.6	20	20	[39]
*Lavandula intermedia*	12.6	15	25	[62]
*Nelumbo nucifera*	12.9 ± 3.7	N.A. ^c^	10	[63]
*Punica granatum*	15	30	45	[64]
*Abelmoschus esculentus*	16.9	65	85	[65]
*Equisetum arvense*	18	64	128	[66]
*Cestrum nocturnum*	20	8	N.A.	[67]
*Vaccinium corymbosum*	20	8	N.A.	[68]
*Carya illinoinensis*	20.34 ± 1.69	16	128	[69]
*Galega officinalis*	23	5	50	[70]
A-BG-AgNPs	16.4 ± 4.9	4	4	Our study
E-BG-AgNPs	9.6 ± 3.7	4	16	Our study

^a^ *E. coli*. ^b^ *S. aureus*. ^c^ data is not provided from the study.

## Data Availability

Not applicable.
